# Clinical implications of head trauma in frontotemporal dementia and primary progressive aphasia

**DOI:** 10.1186/s13195-024-01553-1

**Published:** 2024-08-29

**Authors:** Breton M. Asken, Jessica M. Bove, Russell M. Bauer, Jeremy A. Tanner, Kaitlin B. Casaletto, Adam M. Staffaroni, Lawren VandeVrede, Michael L. Alosco, Jesse B. Mez, Robert A. Stern, Bruce L. Miller, Lea T. Grinberg, Adam L. Boxer, Maria Luisa Gorno-Tempini, Howie J. Rosen, Gil D. Rabinovici, Joel H. Kramer

**Affiliations:** 1 Department of Clinical and Health Psychology, University of Florida, 1Florida Alzheimer’s Disease Research Center, Fixel Institute for Neurological Diseases, PO Box 100165, Gainesville, FL 32610 USA; 2 Department of Neurology, Biggs Institute for Alzheimer’s and Neurodegenerative Diseases South Texas Alzheimer’s Disease Research Center, University of Texas Health – San Antonio, 7703 Floyd Curl Drive, San Antonio, TX 78229 USA; 3 Department of Neurology, Weill Institute for Neurosciences Memory and Aging Center, University of California, San Francisco, UCSF Alzheimer’s Disease Research Center, 675 Nelson Rising Lane, San Francisco, CA 94158 USA; 4 Department of Neurology, Boston University, Boston University Alzheimer’s Disease Research Center and CTE Center, 73 E. Concord Street, Boston, MA 02118 USA

**Keywords:** Frontotemporal dementia, Primary progressive aphasia, Repetitive head impacts, Traumatic brain injury, Risk factor

## Abstract

**Background:**

Traumatic brain injury (TBI) and repetitive head impacts (RHI) have been linked to increased risk for multiple types of neurodegenerative disease, higher dementia risk, and earlier age of dementia symptom onset, suggesting transdiagnostic implications for later-life brain health. Frontotemporal dementia (FTD) and primary progressive aphasia (PPA) represent a spectrum of clinical phenotypes that are neuropathologically diverse. FTD/PPA diagnoses bring unique challenges due to complex cognitive and behavioral symptoms that disproportionately present as an early-onset dementia (before age 65). We performed a detailed characterization of lifetime head trauma exposure in individuals with FTD and PPA compared to healthy controls to examine frequency of lifetime TBI and RHI and associated clinical implications.

**Methods:**

We studied 132 FTD/PPA (age 68.9 ± 8.1, 65% male) and 132 sex-matched healthy controls (HC; age 73.4 ± 7.6). We compared rates of prior TBI and RHI (contact/collision sports) between FTD/PPA and HC (chi-square, logistic regression, analysis of variance). Within FTD/PPA, we evaluated associations with age of symptom onset (analysis of variance). Within behavioral variant FTD, we evaluated associations with cognitive function and neuropsychiatric symptoms (linear regression controlling for age, sex, and years of education).

**Results:**

Years of participation were greater in FTD/PPA than HC for any contact/collision sport (8.5 ± 6.7yrs vs. 5.3 ± 4.5yrs, *p* = .008) and for American football (6.2yrs ± 4.3yrs vs. 3.1 ± 2.4yrs; *p* = .003). Within FTD/PPA, there were dose-dependent associations with earlier age of symptom onset for TBI (0 TBI: 62.1 ± 8.1, 1 TBI: 59.9 ± 6.9, 2 + TBI: 57.3 ± 8.4; *p* = .03) and years of American football (0yrs: 62.2 ± 8.7, 1-4yrs: 59.7 ± 7.0, 5 + yrs: 55.9 ± 6.3; *p* = .009). Within bvFTD, those who played American football had worse memory (z-score: -2.4 ± 1.2 vs. -1.4 ± 1.6, *p* = .02, *d* = 1.1).

**Conclusions:**

Lifetime head trauma may represent a preventable environmental risk factor for FTD/PPA. Dose-dependent exposure to TBI or RHI influences FTD/PPA symptom onset and memory function in bvFTD. Clinico-pathological studies are needed to better understand the neuropathological correlates linking RHI or TBI to FTD/PPA onset and symptoms.

**Supplementary Information:**

The online version contains supplementary material available at 10.1186/s13195-024-01553-1.

## Introduction

Traumatic brain injury (TBI) is associated with increased risk for diagnosis of multiple forms of dementia and with an earlier age of symptom onset than those without prior head trauma [1–4]. Studies supporting these links typically utilize large epidemiological cohorts and leverage medical record diagnostic codes both for determining prior TBI and dementia diagnosis [5]. Alzheimer’s disease is a common focus in TBI-dementia research with some data supporting more severe Alzheimer’s pathology in older adults with prior TBI [6]. Less attention has been given to TBI and the risk for frontotemporal dementia (FTD) or primary progressive aphasia (PPA), a spectrum of conditions characterized by prominent changes in behavior, language, and/or movement in the setting of degeneration of the frontal and temporal lobes, which collectively are the most common form of dementia in individuals under age 65 [7].

Beyond TBI, we now appreciate that the environmental exposure to repetitive head impacts (RHI), regardless of a diagnosed TBI, from contact/collision sports, military service-related blast exposure, or other sources (e.g., intimate partner violence) can independently contribute to later-life dementia risk [8, 9]. RHI is the only known risk factor for chronic traumatic encephalopathy (CTE), a neurodegenerative tauopathy highly specific to prior RHI [8]. The clinical syndrome associated with CTE, known as traumatic encephalopathy syndrome (TES), includes symptoms that can resemble those in FTD (e.g., executive dysfunction, neurobehavioral dysregulation) as well as less common FTD-related symptoms like memory loss [10]. Unfortunately, RHI is not routinely documented in medical records nor well-characterized in large aging-focused studies where deeper clinical phenotyping would offer significant advantages over medical record diagnostic codes. There is therefore limited understanding of environmental factors like TBI or RHI that may be associated with an FTD or PPA diagnosis or the significant variability in symptoms.

Comprehensive and sensitive measures for accurately identifying (and ruling out) TBI with loss of consciousness or posttraumatic amnesia (LOC/PTA) suggest an expected lifetime frequency of ~ 30–35% among older adults [11, 12], with higher rates in men than women. Prior head trauma and FTD research suggests that TBI may be overrepresented in patients with FTD and associated with up to 3 years earlier age of symptom onset among those with the behavioral variant of FTD [1, 13]. However, studies with reported TBI frequencies ranging from 4 to 19% in patients with FTD suggest high likelihood of significant TBI underreporting or misidentification [1, 13–16]. There are no data available on RHI exposure in FTD or PPA. However, studies of brain donors with substantial RHI during life suggest high rates of mixed neuropathology and, in particular, increased risk for tau and TDP-43 accumulation, the most common proteinopathies present in FTD and most forms of PPA [8, 17].

The common finding of multiple neuropathological changes in brain donors with dementia [18–20], and the potential association of head trauma with mixed neuropathology [8, 17, 21], underscores the need for continued in vivo and clinical investigations of head trauma across the dementia spectrum. Identifying the frequency of prior head trauma in FTD and PPA and the relation to clinical features like symptom severity and age of symptom onset has implications for disease prevention, delay, or modification. These factors are especially relevant for early age of onset conditions like FTD or PPA that carry substantial social and economic burden and that pose unique challenges to affected families [22, 23].

We performed a detailed assessment of both TBI and RHI among a well-characterized group of individuals with FTD/PPA spectrum diagnoses compared to clinically normal controls. Our main goals were to better understand head trauma reporting rates in FTD/PPA, whether the type and/or extent of head trauma differed between FTD/PPA and controls, and associations with clinical measures (age of onset, cognitive and neuropsychiatric function).

## Methods

### Participants

Participants were included from the University of California, San Francisco Memory and Aging Center Alzheimer’s Disease Research Center (ADRC), Program Project Grant (PPG) on frontotemporal dementia, or Brain Aging Network for Cognitive Health (BrANCH; healthy controls). ADRC and PPG participants are primarily clinic-based referrals, while BrANCH participants are primarily community-based older adults. All participants underwent detailed neurological and neuropsychological evaluations and diagnosis by multidisciplinary consensus conference. We focused analyses on participants diagnosed by multidisciplinary consensus conference with FTD or PPA spectrum clinical phenotype using published criteria: behavioral variant frontotemporal dementia (bvFTD) [24], logopenic variant primary progressive aphasia (lvPPA) [25], nonfluent variant PPA (nfvPPA) [25], semantic variant PPA (svPPA) [25], cortical basal syndrome (CBS) [26], or a progressive supranuclear palsy syndrome (PSP-S) [27]. Alzheimer’s disease is the most common cause of lvPPA and is the primary etiology in a subset of patients with CBS; we opted to include these clinically-defined diagnostic groups given the lack of existing data on head trauma exposure and their general categorization as “atypical” and often early-onset forms of dementia.

We attempted to age- and sex-match (1:1) the participants with FTD/PPA to community-dwelling healthy controls (HC) characterized as clinically normal, functionally independent, without cognitive concerns. Data from detailed head trauma surveys were not available for participants during multidisciplinary consensus and therefore would not have factored into diagnosis.

### Head trauma exposure assessment

Study participants completed comprehensive evaluations of lifetime head trauma exposure including symptomatic TBI (Ohio State University TBI Identification) and exposure to repetitive head impacts (RHI) from contact and collision sports (Boston University Head Impact Exposure Assessment; see Additional File 1). Surveys were sent to actively enrolled participants to complete independently and data were then linked to the most recent visit. We required loss of consciousness (LOC) or posttraumatic amnesia (PTA) to count towards the prior TBI total. TBI severity was defined by LOC duration as mild (LOC 0–30 min) or moderate-to-severe (LOC > 30 min). RHI through contact/collision sports included participation in American football, boxing, ice hockey, wrestling, rugby, karate/mixed martial arts, lacrosse, and/or soccer. Prior TBI was characterized as “any” (1+) prior TBI and multiple (2+) TBIs. RHI was characterized as “any” prior RHI (dichotomous groups with and without participation in a contact/collision sport for any duration) and as a continuous variable (summed cumulative self-reported total years of participation for each contact/collision sport). We also evaluated RHI via participation in American football, specifically, based on any participation, total years of participation, and exposure thresholds established by recent consensus recommendations for at least “substantial” exposure (5 + years of participation) [10].

We evaluated differences between FTD/PPA and HC in both type and extent of prior head trauma (TBI and RHI separately), the association of prior head trauma with age of symptom onset (FTD/PPA only), and the association of prior head trauma with cognitive and neuropsychiatric symptoms in bvFTD, our largest clinical phenotype group. Sample sizes were insufficient for studying other clinical phenotypes in isolation.

Ages of TBI and RHI were collected through the surveys and adjusted to ensure that only exposures that preceded clinical outcomes (e.g., age of symptom onset) counted towards a participant’s exposure characterization. For example, if reported age of symptom onset was 55, and a TBI was reported to have occurred at age 60, this TBI was subtracted from their TBI total.

### Clinical outcomes

Age of symptom onset for participants in the FTD/PPA group was estimated based on report by the participant and their study partner. For participants with bvFTD, cognitive composite scores were derived from comprehensive neuropsychological assessments [28]. Raw test scores were converted to z-scores relative to the performance of a large, clinically normal reference group (97% with CDR-SB = 0, age 65 ± 13 years, 60% female, education 16.8 ± 2.4 years). Composite z-scores were calculated as the average z-score across tasks within domains of memory (California Verbal Learning Test, Short Form total immediate recall, short delay, and long delay; Benson figure delayed recall), executive function (D-words/one minute, Delis-Kaplan Executive Function Scale Design Fluency Trial 1, Stroop inhibition, longest backward digit span, modified trail making test lines per minute), language (animals/one minute, 15-item Boston Naming Test), and visuospatial (Benson figure copy, Visual Object and Space Perception Number Location). Neuropsychiatric symptoms were quantified with the Neuropsychiatric Inventory questionnaire (NPI-Q).

### Statistical analysis

Frequency of head trauma exposure was compared between HC and FTD/PPA groups using chi-square for categorical classifications and analysis of variance (ANOVA) for continuous head trauma exposure variables (e.g., duration of RHI exposure). Logistic regression with odds ratios were used to assess the association of duration of RHI with odds of FTD/PPA diagnosis. Within the FTD/PPA group, age of symptom onset was compared between participants with and without prior head trauma using ANOVA and stepwise trends based on exposure groups (e.g., 0, 1, 2 + TBIs) were assessed using the chi square linear-by-linear association test. The association with years of RHI (continuous) was evaluated using linear regression. Within the bvFTD group, the association of head trauma with cognitive performance (composite domain z-scores) and neuropsychiatric symptoms (NPI-Q total score) was assessed with analysis of covariance controlling for age, sex, and years of education. We log transformed dependent variables that significantly deviated from a normal distribution and set an a priori alpha of *p* < .05 for defining statistical significance. Effect sizes were calculated for group comparisons and converted to a Cohen’s *d* scale for interpretation (small = 0.20–0.49, medium = 0.50–0.79, large *≥* 0.80) [29].

## Results

Our study cohort included 132 HC and 132 sex-matched FTD/PPA (65.2% male; Table 1). Despite attempts to age-match, the HC group was older than FTD/PPA (73.4 ± 7.6yrs vs. 68.9 ± 8.1, *p* < .001) and reported more completed years of education (17.5 ± 2.0 vs. 16.2 ± 2.5, *p* < .001). In this final study cohort, sport participation history determined via the BU-RHIEA was completed in 100% of participants, while details of prior TBI with LOC or PTA via OSU TBI-ID was completed in 242 (92%) participants with largely preserved sex-matching (HC: 65% male, FTD/PPA: 62% male; *p* = .7). The most common clinical syndrome represented within the FTD/PPA group was bvFTD (*N* = 39, 30%) followed by lvPPA (*N* = 24, 18%), nfvPPA (*N* = 23, 17%), PSP-S and svPPA (Ns = 16, 12% each), and CBS (*N* = 14, 11%).

**Table 1:** Cohort descriptive statistics

	Overall	Healthy Control	FTD/PPA
**N**	264	132	132^e^
**Age**	71.1 ± 8.2	73.4 ± 7.6	68.8 ± 8.1
**Sex**,** N(%) Female**	92 (34.8)	46 (34.8)	46 (34.8)
**Education**,** yrs**	16.9 ± 2.3	17.5 ± 2.0	16.2 ± 2.5
**Race**,** N(%)**			
*White*	226 (89.3)	114 (87.0)	112 (91.8)
*Asian* ^*a*^	15 (6.0)	11 (8.5)	4 (3.2)
*Black*	7 (2.8)	3 (2.3)	4 (3.3)
*Unknown/Refused*	5 (2.0)	3 (2.3)	2 (1.6)
**TBI**^**b**^, **N(%male) / N(%female)**			
*0*	90 (58.8) / 66 (74.2)	51 (60.7) / 32 (69.6)	39 (56.5) / 34 (79.1)
*1*	36 (23.5) / 16 (18.0)	22 (26.2) / 9 (19.6)	14 (20.3) / 7 (16.3)
*2+*	27 (17.6) / 7 (7.9)	11 (13.1) / 5 (10.9)	16 (23.2) / 2 (4.7)
**RHI** **N(%male) / N(%female)**			
Any RHI Exposure^c^	87 (50.6) / 7 (7.6)	38 (44.2) / 6 (13.0)	49 (57.0) / 1 (2.2)
*Am. Football*	59 (34.3) / 0 (0.0)	27 (31.4) / 0 (0.0)	32 (37.2) / 0 (0.0)
*Boxing*	8 (4.7) / 0 (0.0)	3 (3.5) / 0 (0.0)	5 (5.8) / 0 (0.0)
*Soccer*	28 (16.3) / 7 (7.6)	14 (16.3) / 6 (13.0)	14 (16.3) / 1 (2.2)
*Ice Hockey*	6 (3.5) / 0 (0.0)	1 (1.2) / 0 (0.0)	5 (5.8) / 0 (0.0)
*Karate / Mixed Martial Arts*	2 (1.2) / 0 (0.0)	1 (1.2) / 0 (0.0)	1 (1.2) / 0 (0.0)
*Wrestling*	8 (4.7) / 0 (0.0)	2 (2.3) / 0 (0.0)	6 (7.0) / 0 (0.0)
*Rugby*	2 (1.2) / 0 (0.0)	1 (1.2) / 0 (0.0)	1 (1.2) / 0 (0.0)
*Lacrosse*	1 (0.6) / 0 (0.0)	1 (1.2) / 0 (0.0)	0 (0.0) / 0 (0.0)
Cumulative exposure, yrs^d^	6.6 ± 5.6 / 9.1 ± 8.4	5.1 ± 4.5 / 6.5 ± 5.1	8.0 ± 6.2 / 25 (*n* = 1)
*Am. football exposure*,* yrs*	4.7 ± 3.8 / na	3.1 ± 2.4 / na	6.2 ± 4.3 / na
Am. Football (Highest Level of Play*)*			
*Professional*	1 (0.6) / 0 (0.0)	0 (0.0) 0 (0.0)	1 (1.2) / 0 (0.0)
*College*	10 (5.8) / 0 (0.0)	4 (4.7) / 0 (0.0)	6 (7.0) / 0 (0.0)
*High School*	38 (22.1) / 0 (0.0)	18 (20.9) / 0 (0.0)	20 (23.3) / 0 (0.0)
*Youth*	8 (4.7) / 0 (0.0)	4 (4.7) / 0 (0.0)	4 (4.7) / 0 (0.0)
*Unknown*	2 (1.2) / 0 (0.0)	1 (1.2) / 0 (0.0)	1 (1.2) / 0 (0.0)

^a^ Inclusive of Indian, Chinese, Japanese, and “other” Asian descent

^b^ 242/264 participants completed the OSU TBI-ID for TBI history (153 male, 89 female). For HC, 2 (1.5%) and for FTD/PPA, 6 (5.4%) participants reported a prior TBI considered moderate-to-severe based on LOC > 30 min

^c^ Total N across individual sports will exceed N of “Any RHI Exposure” due to instances of multi-sport participation

^d^ Sum of self-reported number of years participating in each of American football, boxing, soccer, ice hockey, karate/mixed martial arts, wrestling, rugby, or lacrosse

^e^*N* = 13 FTD/PPA participants with known pathogenic variants in *C9orf72* (*N* = 5), *GRN* (*N* = 4), *MAPT* (*N* = 2), or *TARDBP* (*N* = 2). No HC participants had FTLD-related pathogenic variants

### Type and extent of head trauma exposure

History of any TBI did not significantly differ between HC (36.2%) and FTD/PPA (34.8%), nor did frequency of multiple (2+) TBIs (HC: 12.3%, FTD/PPA: 16.1%; *p* = .51). Prior moderate-severe TBI was rare in both groups but qualitatively higher in FTD/PPA than HC (5.4% vs. 1.5%, *p* = .15). Between-group results were similar when examining males and females separately.

**Fig. 1:**
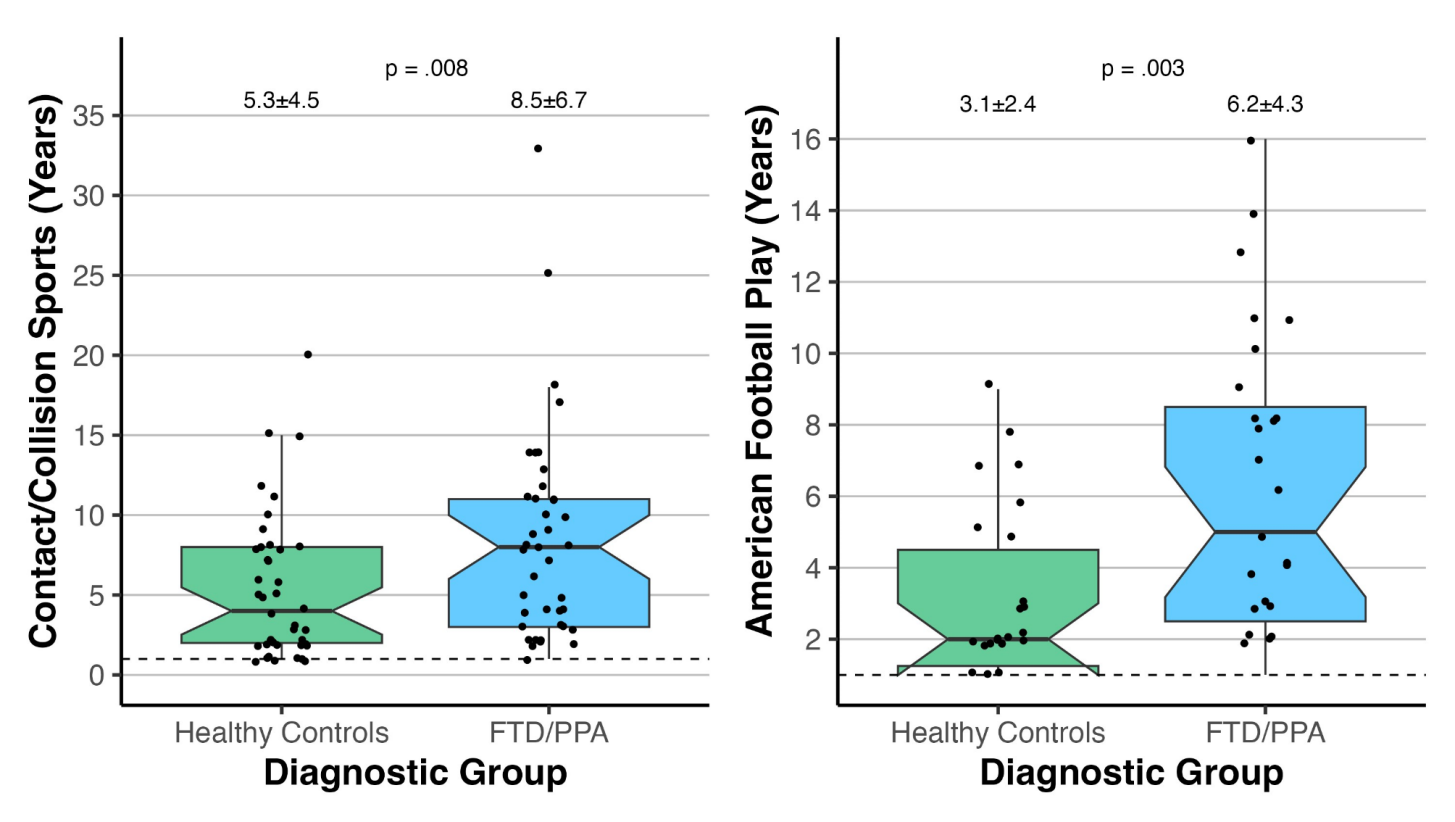
Differences in duration contact/collision sport (LEFT) and American football (RIGHT) participation between Healthy Controls (*N* = 41) and FTD/PPA (*N* = 40) reporting at least 1 year of play (i.e., not including those who never participated). The dashed line represents the minimum duration of participation (1 year). Duration of contact/collision sport exposure was longer in FTD/PPA than HC (8.5 ± 6.7yrs vs. 5.3 ± 4.5yrs, *p* = .008). For American football, participants with FTD/PPA played about twice as long as HC (6.2yrs ± 4.3yrs vs. 3.1 ± 2.4yrs; *p* = .003)

Frequency of any prior participation in a contact/collision sport did not differ between HC and FTD/PPA (33.3% vs. 37.9%; *p* = .60), including American football (20.5% vs. 24.2%; *p* = .46). However, among those who previously participated in contact/collision sports (FTD/PPA *N* = 40, HC *N* = 41 with known duration data), duration of RHI exposure was longer in FTD/PPA than HC (8.5 ± 6.7yrs vs. 5.3 ± 4.5yrs, *p* = .008; Fig. 1). This effect appeared driven by American football wherein participants with FTD/PPA played about twice as long as HC (6.2yrs ± 4.3yrs vs. 3.1 ± 2.4yrs; *p* = .003). Participants with FTD/PPA who previously played American football were twice as likely to have played for 5 + years compared to HC, though this effect did not reach statistical significance (51.9% vs. 26.9%, *p* = .06).

We identified a significant interaction effect of age and duration of American football exposure on the odds of FTD/PPA (*p* = .02). Duration of American football participation was associated with higher odds of FTD/PPA among younger participants only. To further probe this effect, we examined odds ratios along the age spectrum and saw this interaction effect represented when stratifying analyses by participants at age 75. Among participants below 75, each additional year of American football exposure was associated with 36% greater odds of FTD/PPA (OR = 1.36 [1.01–1.83], *p* = .04 controlling for age, sex, and education) while an opposite effect was seen in participants age 75 or older (OR = 0.77 [0.54–1.08], *p* = .13).

Additional File 2 – Table 1 shows TBI and RHI history for each diagnostic group within FTD/PPA.

### Head Trauma exposure and age of Symptom Onset

**Fig. 2:**
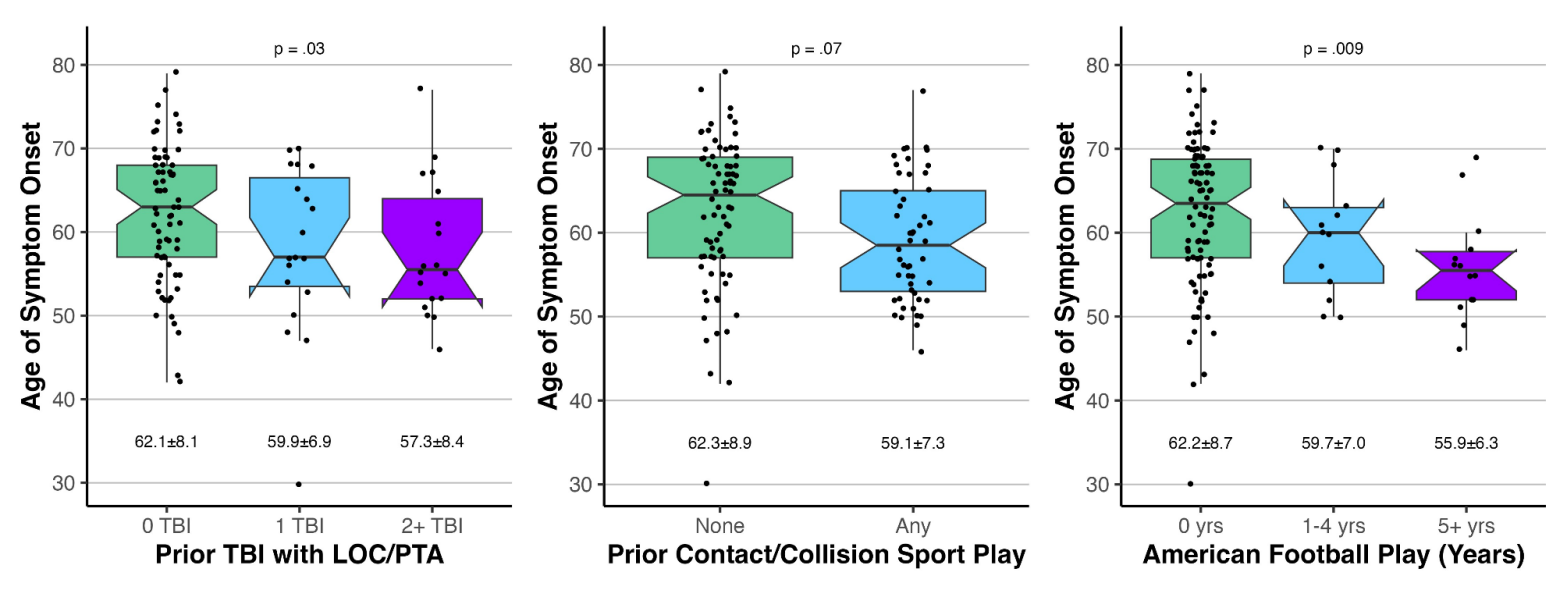
Association between age of symptom onset and prior head trauma exposure among participants with FTD/PPA. Differences in age of symptom onset are shown as a function of prior TBI (left), prior participation in contact/collision sport (middle), and prior participation in American football (right). Group-specific age of symptom onset is displayed within each figure (mean ± standard deviation). Alpha levels (p values) provided for 3 group comparisons represent the chi square linear-by-linear association test evaluating statistical significance for a stepwise effect across the 3 groups

Participants with FTD/PPA and any prior TBI evidenced a younger age of symptom onset than those without prior TBI with a medium effect size that did not reach statistical significance (58.5 ± 7.7 vs. 62.1 ± 8.1, *p* = .054, *d* = 0.45). There was a stepwise progression of earlier onset with multiple (2+) TBI (1 TBI = 59.9 ± 6.9, 2 + TBI = 57.3 ± 8.4, *p* = .03, Fig. 2). Sex did not significantly moderate this association, though there were relatively few females with FTD/PPA and prior TBI with known age of symptom onset (*N* = 7). Results were similar when excluding 2 individuals whose TBI reportedly occurred within 5 years of symptom onset.

Prior participation in contact/collision sports was associated with younger age of symptom onset and small-medium effect size that did not reach statistical significance (59.1 ± 7.3 vs. 62.3 ± 8.9, *p* = .07, *d* = 0.40; Fig. 2). A stronger effect was seen when examining American football participation (57.7 ± 6.9 vs. 62.2 ± 8.7, *p* = .02, *d* = 0.54) with a stepwise decrease in age of symptom onset with greater duration of exposure (1-4yrs: 59.7 ± 7.0, 5 + yrs: 55.9 ± 6.3, *p* = .009). Among participants with any American football participation, there was some evidence for a dose-dependent effect of more years of American football play with younger age of symptom onset, but this was not statistically significant (β=-0.31, *p* = .11). Given that bvFTD may, on average, have an earlier age of symptom onset than other FTD/PPA conditions and be more likely to affect males (thus potentially inherent higher rates of head trauma among a condition with earlier age of symptom onset), we repeated analyses excluding those with bvFTD and results were unchanged. Age of symptom onset for each clinical diagnostic group is provided in Additional File 2 – Table 2.

Given the expected disproportionate male exposure to TBI and RHI, we wanted to better understand whether the observed age of onset differences associated with prior head trauma could instead be capturing a broader effect of sex on age of symptom onset. To do so, we restricted analysis to participants with FTD/PPA who denied history of both prior TBI and RHI (*N* = 23 males, *N* = 32 females), and there was no difference in age of symptom onset between males and females (62.0 ± 7.9 vs. 63.4 ± 10.0, *p* = .59, *d* = 0.15).

### Cognition and neuropsychiatric symptoms in bvFTD

There were 26 participants with bvFTD with established TBI history (*N* = 9 with 1 + TBI) and 32 with established RHI history (*N* = 16 with any prior contact/collision sport participation, *N* = 10 with prior American football participation) who also completed the NPI-Q. Neuropsychiatric symptoms (NPI-Q total score) were higher in those *without* prior TBI (49.2 ± 14.7 vs. 24.7 ± 19.3, *p* = .001, *d* = 1.5). The opposite was seen when stratifying by American football, where neuropsychiatric symptoms were higher in bvFTD participants who played American football than those who did not, but the difference was not statistically significant (NPI-Q total scores: 47.7 ± 11.6 vs. 38.1 ± 23.5, *p* = .18, *d* = 0.36). Comparisons across NPI-Q domain subscores are provided in Additional File 2 – Table 3.

**Fig. 3:**
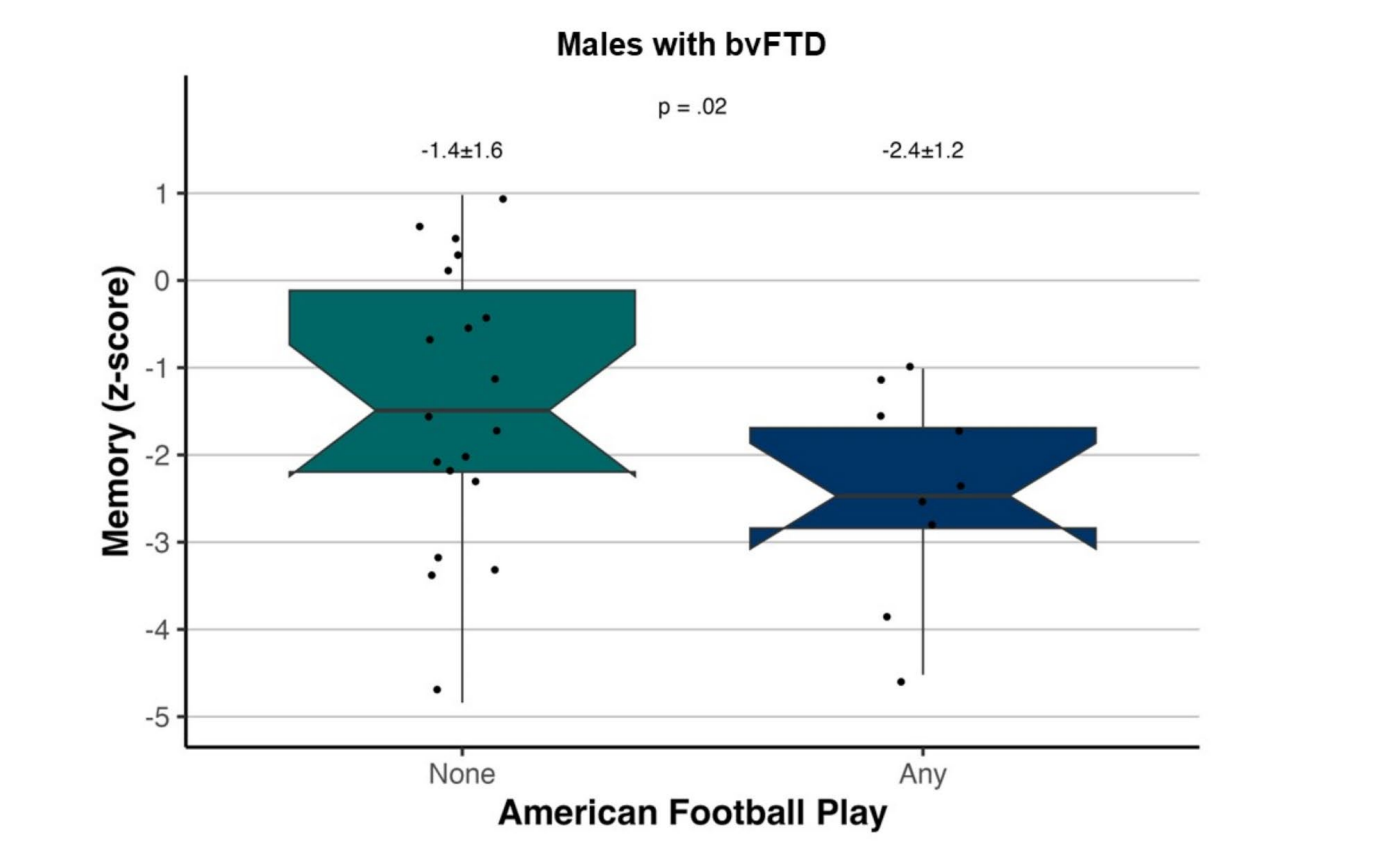
Association of prior American football participation with objective memory test scores among individuals with behavioral variant frontotemporal dementia (bvFTD). Objective memory scores were significantly lower among those who had played American football

There were 23 participants with bvFTD with established TBI history (*N* = 6 with 1 + TBI) and 28 with established RHI history (*N* = 15 with any prior contact/collision sport, *N* = 9 with prior American football) who also completed comprehensive neuropsychological testing. Cognitive test scores did not differ between bvFTD with and without any prior TBI or with and without any RHI. Conversely, participants with bvFTD who previously played American football had lower memory scores (z-score: -2.4 ± 1.2 vs. -1.4 ± 1.6, *p* = .02, *d* = 1.1; Fig. 3) but did not differ in other cognitive domains.

## Discussion

We describe lifetime head trauma exposure spanning both TBI with LOC/PTA and RHI through contact/collision sports in a well-characterized cohort of individuals with FTD and PPA syndromes. Our comprehensive approach to surveying lifetime head trauma exposure provides more accurate estimates of head trauma in FTD/PPA, as well as the type and extent of head trauma that differ between FTD/PPA and clinically normal older adults.

We found that duration of RHI exposure, predominantly in American football, was significantly longer in FTD/PPA than healthy controls. Longer RHI duration appeared most related to increased odds of FTD/PPA diagnosis for younger individuals in our cohort (early 70s and younger). We additionally observed a dose-response exposure effect where more frequent TBI and longer duration of RHI exposure with earlier age of symptom onset among FTD/PPA. Additionally, in participants with bvFTD, prior RHI through American football related to worse objective memory testing than those with bvFTD who had not previously played American football. History of any TBI or multiple TBI did not differ between FTD/PPA and healthy controls, nor did the frequency of having participated at some point in contact/collision sports. The findings emphasize the relatively high frequency of head trauma among older adults generally, that greater duration of RHI through contact/collision sports may constitute an environmental risk factor for FTD/PPA diagnosis, and the potential influence of head trauma on clinical variability observed among patients diagnosed with FTD/PPA.

We observed rates of prior TBI with LOC/PTA exceeding 30–40% across study groups, which is significantly higher than reported head trauma in prior FTD studies [1, 14]. These findings are likely associated with improved sensitivity of the OSU TBI-ID over cursory screening measures or medical record coding, as previously reported [11]. RHI through contact/collision sports has not been reported in prior FTD/PPA work and had not been systematically documented for our participants prior to this study. Using the BU-HIEA, we identified that a significant proportion of FTD/PPA males (> 40%) previously played a contact/collision sport, including more than a third of males who played American football at some level.

Consistent with the hypothesis that duration of RHI is a key risk factor later-life neurodegeneration, particularly CTE [30–32], participants with FTD/PPA reported almost twice the number of years of contact/collision sport play and American football participation reported by healthy controls. Duration of American football play was around 3 years, on average, for healthy controls versus 6 years for FTD/PPA. This may lend support to recently proposed thresholds of 5 + years constituting “substantial” or clinically meaningful RHI exposure [10, 30]. Earlier age of symptom onset was also observed in FTD/PPA adults with American football participation, with the youngest average age of onset among those who played more than 5 years. Our study lacked neuropathological characterization of study participants and most of the suspected proteinopathies relevant to our sample (e.g., CTE, FTLD tau or TDP43) do not have validated in vivo biomarkers. Future clinico-pathological studies are needed to determine neuropathological correlates of RHI in this cohort (e.g., presence of CTE, more severe FTLD-related or other neuropathology, higher rates of mixed neuropathology). This is especially important for etiologically heterogenous clinical phenotypes like bvFTD and for diseases like CTE where the associated clinical phenotype remains less well developed.

In general, RHI exposure and duration more consistently differed between FTD/PPA and healthy controls and more strongly related to clinical measures than prior TBI with LOC/PTA. Our group and others have shown that medial temporal structures are especially susceptible to the neurodegenerative effects of RHI [17, 33, 34], which is consistent with the specific association observed between RHI and worse memory in our subgroup of participants with bvFTD. Memory loss is a core clinical feature of traumatic encephalopathy syndrome, the clinical phenotype associated with underlying CTE pathology and/or other neuropathological consequences of substantial RHI [8, 10, 17, 33, 35]. Traditionally, memory is thought to be relatively preserved in bvFTD or, when impaired, reflects indirect effects of executive dysfunction. In many cases memory loss might be considered exclusionary for diagnosing bvFTD [24, 36]. Others have argued reasonably that a subset of patients with bvFTD have impaired memory and presence of memory loss should not disqualify a bvFTD diagnosis [37, 38]. While preliminary, our findings suggest that prior RHI may partially explain why memory loss is observed in some, but not all, patients with bvFTD.

Many factors contribute to heterogeneity in clinical syndromes, age of symptom onset, and specific symptoms in individuals with neurodegenerative diseases. Regarding FTD and early-onset atypical (nonamnestic) forms of Alzheimer’s disease, life-course risk factors have been identified previously, such as higher rates of dyslexia in patients with lvPPA [39] and higher rates of mathematical or visuospatial learning difficulties in patients with posterior cortical atrophy [40]. It is possible that substantial head trauma exposure contributes to brain network vulnerability and influences risk for and progression of neurodegenerative diseases [41, 42]. This may be particularly true for individuals with atypical presentations (e.g., memory loss in bvFTD), or have earlier symptom onset, which is especially relevant in genetic forms of FTLD where there is significant emphasis on understanding factors that relate to symptom onset timing.

Strengths of our study were the comprehensive assessments of head trauma and the clinical phenotyping supporting our diagnostic groups. Characterizing lifetime head trauma is retrospective by nature and limited by self-report. Age of symptom onset also relied on retrospective self-report. Participants with cognitive decline, especially language-predominant difficulties, may be biased towards underreporting of prior head trauma; however, the similar or higher rates of head trauma observed in the FTD/PPA group compared to HC somewhat alleviates these concerns. FTD/PPA participants were recruited from a specialty dementia clinic while HC were community-based. RHI through earlier life participation in contact/collision sports like American football overwhelmingly affects older adult males more than females and more research in females with prior RHI is needed. RHI exposure is not a uniform exposure variable. There are differences in frequency and magnitude of head impacts sustained by different playing positions or styles of play, and future work should continue refining RHI as a proxy measure for head trauma exposure [31]. We were underpowered to appropriately study whether there were sex-specific effects of TBI on clinical measures within the FTD/PPA group or to examine associations between head trauma and clinical measures within all FTD/PPA phenotypes. We required TBI accompanied by LOC or PTA, which likely increases specificity to prior symptomatic head trauma, but also lacks sensitivity to most TBI classified as “concussion” not involving LOC or PTA, as is often the case in sport and recreation settings. Neuropsychiatric symptoms were derived from a single measure (NPI-Q total score). More detailed assessment of behavioral and psychiatric symptoms is needed to understand symptoms that may differ because of prior head trauma versus being common to FTD. This study sample lacked racial and ethnic diversity and we cannot generalize findings to older adults that do not identify as non-Hispanic/white. Additional work emphasizing recruitment of older adults from underrepresented backgrounds is important given potentially higher rates of both head trauma exposure and dementia diagnosis [43].

## Conclusions

Lifetime head trauma exposure is prevalent among older adults, and both TBI and RHI exposure should be documented routinely in clinical and research settings for neurodegenerative diseases. Participants with FTD/PPA have significantly longer duration of RHI exposure through contact/collision sports like American football compared to cognitively unimpaired, healthy adults. Dose-dependent exposure to TBI or RHI relates to earlier age of FTD/PPA symptom onset. Lifetime head trauma, especially RHI, may represent a preventable environmental risk factor for FTD/PPA. Clinico-pathological studies are needed to better understand the neuropathological correlates linking TBI and RHI to FTD/PPA onset and symptoms.

## Electronic supplementary material

Below is the link to the electronic supplementary material.

Supplementary Material 1: Additional file 1-Supplemental Methods.

Supplementary Material 2: Additional file 2-Supplemental Results.

## Data Availability

All study data are available on reasonable request made to the UCSF Memory and Aging Center. Academic, not-for-profit investigators can request data for professional education and for research studies. Requests can be made online (https://memory.ucsf.edu/research-trials/professional/open-science). Datasets used for the analyses for the current study are also available from the corresponding author on reasonable request.

## Abbreviations

ADRC: Alzheimer’s Disease Research Center
ANOVA: Analysis of variance
bvFTD: Behavioral variant frontotemporal dementia
CBS: Corticobasal syndrome
CTE: Chronic traumatic encephalopathy
FTD: Frontotemporal dementia
HC: Healthy controls
LOC: Loss of consciousness
lvPPA: Logopenic variant primary progressive aphasia
nfvPPA: Nonfluent/agrammatic variant primary progressive aphasia
NPI: Q–Neuropsychiatric Inventory Questionnaire
PPA: Primary progressive aphasia
PPG: program project grant
PSP: S–progressive supranuclear palsy syndrome
PTA: Posttraumatic amnesia
RHI: Repetitive head impacts
svPPA: Semantic variant primary progressive aphasia
TBI: Traumatic brain injury
TES: Traumatic encephalopathy syndrome
