# Supplementary material for: Clinical implications of head trauma in frontotemporal dementia and primary progressive aphasia
Source: Alzheimers Res Ther. 2024 Aug 29;16:193. doi: 10.1186/s13195-024-01553-1 (PMC11363650; doi:10.1186/s13195-024-01553-1)
Supplement: Supplementary file 1 — Supplementary Material 1: Additional file 1-Supplemental Methods. [file 13195_2024_1553_MOESM1_ESM.pdf]

ADDITIONAL FILE 1 – SUPPLEMENTAL METHODS

Head Trauma Exposure Assessments

TBI history was assessed with the Ohio State University Traumatic Brain Injury Identification method (OSU TBI-ID)(1, 2). The OSU TBI-ID is a two-tiered survey that first asks participants a series of yes-no questions about whether they have been involved in any activities known to carry high risk of head trauma (e.g., motor vehicle crashes, fights, sports). A "yes" response to any question leads to follow-up questions about injury-related details including age of injury, presence or absence of loss of consciousness (LOC) or posttraumatic amnesia (PTA), duration of LOC, and hospitalization status. TBI severity was defined by LOC duration as mild (LOC 0-30 minutes) or moderate-to-severe (LOC > 30 minutes). The OSU TBI-ID allows for determining frequency of lifetime TBI with LOC or PTA, which was the focus of the TBI factor for our study. Prior TBI was characterized as "any" (1+) prior TBI and multiple (2+) TBIs.

RHI was assessed with the Boston University Head Impact Exposure Assessment (BU-HIEA). The BU-HIEA queries history of participation in contact/collision sports, duration of exposure details like ages and number of years of participation, level of play, and sport-specific details (e.g., playing position)(3, 4). Contact/collision sports included participation in American football, boxing, ice hockey, wrestling, rugby, karate/mixed martial arts, lacrosse, and/or soccer. RHI was characterized as "any" prior RHI (dichotomous groups with and without participation in a contact/collision sport for any duration) and as a continuous variable (summed cumulative self-reported total years of participation for each contact/collision sport). We also evaluated RHI via participation in American football, specifically, based on any participation, total years of participation, and exposure thresholds established by recent consensus recommendations for at least "substantial" exposure (5+ years of participation).

1. Corrigan JD, Bogner J. Initial reliability and validity of the Ohio State University TBI Identification Method. J Head Trauma Rehabil. 2007;22(6):318-29.
2. Gardner RC, Rivera E, O'Grady M, Doherty C, Yaffe K, Corrigan JD, et al. Screening for Lifetime History of Traumatic Brain Injury Among Older American and Irish Adults at Risk for Dementia: Development and Validation of a Web-Based Survey. J Alzheimers Dis. 2020;74(2):699-711.
3. Montenigro PH, Alosco ML, Martin BM, Daneshvar DH, Mez J, Chaisson CE, et al. Cumulative Head Impact Exposure Predicts Later-Life Depression, Apathy, Executive Dysfunction, and Cognitive Impairment in Former High School and College Football Players. J Neurotrauma. 2017;34(2):328-40.
4. Bruce HJ, Tripodis Y, McClean M, Korell M, Tanner CM, Contreras B, et al. American Football Play and Parkinson Disease Among Men. JAMA Netw Open. 2023;6(8):e2328644.
