# Supplementary material for: Clinical implications of head trauma in frontotemporal dementia and primary progressive aphasia
Source: Alzheimers Res Ther. 2024 Aug 29;16:193. doi: 10.1186/s13195-024-01553-1 (PMC11363650; doi:10.1186/s13195-024-01553-1)
Supplement: Supplementary file 2 — Supplementary Material 2: Additional file 2-Supplemental Results. [file 13195_2024_1553_MOESM2_ESM.pdf]

ADDITIONAL FILE 2 – SUPPLEMENTAL RESULTS

	N	N	TBI		Contact/Collision Sport		American Football (males only)		
	TBI History	*Contact/ Collision Sport History*	*Any (n, %)*	*Multiple (n, %)*	*Any (n, %)*	*Duration (years)*	*Any (n, %)*	*Duration (years)*	*5+yrs (n, %)*
**HC**	130	132	47 (36.2)	16 (12.3)	36 (41.9)	4.4±3.2	27 (31.4)	3.1±2.4	7 (8.2)
**bvFTD**	31	39	10 (32.3)	4 (12.9)	18 (64.3)	9.8±4.6	12 (42.9)	7.6±4.6	6 (24.0)
**lvPPA**	22	24	10 (45.5)	7 (31.8)	8 (50.0)	8.9±8.8	8 (50.0)	7.1±5.2	4 (25.0)
**nfvPPA**	19	23	4 (21.1)	2 (10.5)	5 (50.0)	6.2±3.4	4 (40.0)	5.8±2.9	3 (10.0)
**svPPA**	14	16	6 (42.9)	1 (7.1)	5 (45.5)	4.0±2.8	3 (27.3)	2.0	0 (0.0)
**CBS**	14	14	5 (35.7)	2 (14.3)	5 (41.7)	4.8±4.3	2 (16.7)	2.5±2.1	0 (0.0)
**PSP-S**	12	16	4 (33.3)	2 (16.7)	4 (44.4)	7.5±7.3	3 (33.3)	3.7±2.1	1 (11.1)

Supplemental Table 1: Type and extent of head trauma exposure for healthy controls (HC) and for each diagnosis within the FTD/PPA group. Data presented as N (percentage) within the diagnosis (males only for American football). The first two data columns show the number of participants within each diagnosis group that completed the Ohio State University TBI Identification Methods to determine TBI history (N, TBI History) and the Boston University Head Impact Exposure Assessment (N, Contact/Collision Sport History).

Abbreviations: bvFTD – behavioral variant frontotemporal dementia, CBS – corticobasal syndrome, HC – healthy controls, lvPPA – logopenic variant primary progressive aphasia, nfvPPA – nonfluent/agrammatic variant primary progressive aphasia, PSP-S – progressive supranuclear palsy syndrome, svPPA – semantic variant primary progressive aphasia

	TBI		Contact/Collision Sport		American Football	
	None	*Any*	*None*	*Any*	*None*	*Any*
**bvFTD**	57.2±9.5	53.8±11.4	57.0±11.9	57.6±7.1	57.8±10.8	56.2±6.9
**lvPPA**	62.3±7.8	57.4±6.8	61.5±7.7	57.6±7.2	62.1±7.7	56.0±5.9
**nfvPPA**	67.7±4.9	55.8±4.2*	66.1±6.8	61.8±5.7*	66.1±6.6	61.0±6.2*
**svPPA**	61.1±4.0	60.2±9.4*	61.7±6.2	56.6±6.5*	61.2±5.6	55.0±8.7*
**CBS**	64.7±6.3	62.6±12.1*	62.6±9.0	65.3±8.2	64.3±8.4	62.0±11.3*
**PSP-S**	62.3±8.3	63.3±5.7*	66.9±5.2	59.2±7.2*	64.5±7.4	63.7±4.7*

* N≤5

Supplemental Table 2: Age of symptom onset associated with prior head trauma exposure for each diagnosis within the FTD/PPA group.

Abbreviations: bvFTD – behavioral variant frontotemporal dementia, CBS – corticobasal syndrome, lvPPA – logopenic variant primary progressive aphasia, nfvPPA – nonfluent/agrammatic variant primary progressive aphasia, PSP-S – progressive supranuclear palsy syndrome, svPPA – semantic variant primary progressive aphasia

Neuropsychiatric Inventory (NPI) Scores						
	TBI		Contact/Collision Sport		American Football	
	None	*Any*	*None*	*Any*	*None*	*Any*
**All FTD/PPA**						
**N**	46	23	53	29	61	21
**Total**	29.2±21.2	17.4±16.0	22.9±19.8	32.3±21.4	24.2±20.2	32.0±21.7
**Delusions**	0.3±1.1	0.1±0.5	0.2±1.0	0.2±0.5	0.2±1.0	0.2±0.6
**Hallucinations**	0.1±0.4	0.1±0.4	0.1±0.3	0.2±0.5	0.1±0.3	0.2±0.5
**Agitation/Aggression**	2.1±2.7	0.7±1.7	1.6±2.3	2.6±3.3	1.7±2.3	2.7±3.6
**Depression**	1.7±2.1	1.0±1.8	1.9±2.5	1.3±2.0	1.7±2.4	1.5±2.2
**Anxiety**	2.6±3.0	1.9±2.9	2.1±2.7	2.3±2.9	2.2±2.8	2.1±2.7
**Elation/Euphoria**	2.0±3.1	1.1±2.1	1.3±2.6	2.4±3.1	1.4±2.6	2.5±3.3
**Apathy/Indifference**	4.7±3.6	2.6±3.0	3.6±3.6	4.6±3.5	3.7±3.6	4.7±3.5
**Disinhibition**	3.8±3.6	2.7±3.5	2.8±3.3	5.1±4.2	3.2±3.5	4.9±4.4
**Irritability**	2.8±3.3	1.8±3.2	2.7±3.5	3.3±3.6	2.9±3.6	3.0±3.2
**Aberrant Motor Behavior**	3.2±4.1	1.7±2.7	2.3±3.4	3.7±4.2	2.5±3.6	3.6±4.1
**Sleep/Nighttime Behavior**	2.2±3.0	1.6±2.7	1.9±2.9	1.8±2.6	1.9±2.9	1.8±2.3
**Appetite/Eating Behavior**	3.6±3.9	2.2±3.1	2.3±3.4	4.9±4.1	2.7±3.6	4.9±4.1
**bvFTD**						
**N**	17	9	16	16	10	22
**Total**	49.2±14.7	24.7±19.3	37.8±25.1	44.4±15.5	38.1±23.5	47.7±11.6
**Delusions**	0.8±1.7	0.2±0.7	0.8±1.8	0.2±0.5	0.5±1.5	0.3±0.7
**Hallucinations**	0.4±0.6	0.2±0.7	0.2±0.5	0.3±0.6	0.2±0.5	0.4±0.7
**Agitation/Aggression**	3.6±3.4	1.7±2.3	3.1±3.0	3.3±3.5	2.8±2.9	4.0±4.0
**Depression**	1.9±2.4	0.1±0.3	1.3±1.7	1.1±2.2	1.1±1.7	1.3±2.5
**Anxiety**	3.5±3.1	2.0±3.2	2.4±2.9	2.6±3.2	2.6±3.1	2.2±2.9
**Elation/Euphoria**	3.8±2.9	1.1±2.3	2.5±3.0	3.4±3.3	2.6±2.8	3.7±3.8
**Apathy/Indifference**	7.4±2.0	3.7±3.8	5.6±3.9	6.6±2.7	5.5±3.9	7.6±0.8
**Disinhibition**	7.1±1.9	4.1±4.3	5.3±3.3	6.9±3.5	5.5±3.5	7.3±3.2
**Irritability**	4.5±4.0	3.4±4.3	4.8±4.7	4.8±3.8	4.8±4.7	4.7±3.1
**Aberrant Motor Behavior**	5.8±4.5	1.9±3.1	4.1±4.4	5.3±4.4	4.4±4.5	5.3±4.3
**Sleep/Nighttime Behavior**	3.6±3.6	2.4±2.2	3.1±3.6	2.8±3.0	3.0±3.5	2.9±2.7
**Appetite/Eating Behavior**	6.8±3.7	3.8±4.1	4.7±4.2	7.3±3.4	5.0±4.2	8.0±2.7

Supplemental Table 3: Neuropsychiatric Inventory (NPI-Q) scores (mean ± standard deviation) among participants with FTD/PPA and the behavioral variant FTD (bvFTD) subset. NPI-Q scores are shown for the total score and each of the 12 domain scores.
